# The diagnostic and prognostic potential of the EGFR/MUC4/MMP9 axis in glioma patients

**DOI:** 10.1038/s41598-022-24099-4

**Published:** 2022-11-18

**Authors:** Agathe Quesnel, Nathan Coles, Tuomo M. Polvikoski, George S. Karagiannis, Claudio Angione, Meez Islam, Ahmad A. Khundakar, Panagiota S. Filippou

**Affiliations:** 1 School of Health & Life Sciences, Teesside University, Middlesbrough, TS1 3BX UK; 2 National Horizons Centre, Teesside University, 38 John Dixon Ln, Darlington, DL1 1HG UK; 3 Translational and Clinical Research Institute, Newcastle University, Newcastle Upon Tyne, UK; 4 Department of Microbiology and Immunology, Albert Einstein College of Medicine, Bronx, NY USA; 5 Integrated Imaging Program, Albert Einstein College of Medicine, Bronx, NY USA; 6 Gruss-Lipper Biophotonics Center, Albert Einstein College of Medicine, Bronx, NY USA; 7 Tumor Microenvironment and Metastasis Program, Albert Einstein Cancer Center, Bronx, NY USA; 8 School of Computing, Engineering & Digital Technologies, Teesside University, Middlesbrough, UK; 9 Centre for Digital Innovation, Teesside University, Middlesbrough, UK

**Keywords:** Biochemistry, Biological techniques, Cancer, Molecular biology, Systems biology, Biomarkers, Oncology

## Abstract

Glioblastoma is the most aggressive form of brain cancer, presenting poor prognosis despite current advances in treatment. There is therefore an urgent need for novel biomarkers and therapeutic targets. Interactions between mucin 4 (MUC4) and the epidermal growth factor receptor (EGFR) are involved in carcinogenesis, and may lead to matrix metalloproteinase-9 (MMP9) overexpression, exacerbating cancer cell invasiveness. In this study, the role of MUC4, MMP9, and EGFR in the progression and clinical outcome of glioma patients was investigated. Immunohistochemistry (IHC) and immunofluorescence (IF) in fixed tissue samples of glioma patients were used to evaluate the expression and localization of EGFR, MMP9, and MUC4. Kaplan–Meier survival analysis was also performed to test the prognostic utility of the proteins for glioma patients. The protein levels were assessed with enzyme-linked immunosorbent assay (ELISA) in serum of glioma patients, to further investigate their potential as non-invasive serum biomarkers. We demonstrated that MUC4 and MMP9 are both significantly upregulated during glioma progression. Moreover, MUC4 is co-expressed with MMP9 and EGFR in the proliferative microvasculature of glioblastoma, suggesting a potential role for MUC4 in microvascular proliferation and angiogenesis. The combined high expression of MUC4/MMP9, and MUC4/MMP9/EGFR was associated with poor overall survival (OS). Finally, MMP9 mean protein level was significantly higher in the serum of glioblastoma compared with grade III glioma patients, whereas MUC4 mean protein level was minimally elevated in higher glioma grades (III and IV) compared with control. Our results suggest that MUC4, along with MMP9, might account for glioblastoma progression, representing potential therapeutic targets, and suggesting the ‘MUC4/MMP9/EGFR axis’ may play a vital role in glioblastoma diagnostics.

## Introduction

Gliomas include tumors arising from glial cells in the central nervous system and can be classified into four grades (I to IV) according to their histological characteristics (2016 WHO classification). More recently, gliomas have been further classified according to their molecular and cytogenetic characteristics (2021 WHO classification)^1^. Glioblastoma (GBM) is the most common malignant form of brain tumors^2,3^. Standard treatment for GBM includes surgical resection of the tumor, followed by radiotherapy and/or chemotherapy, such as temozolomide. However, almost invariably, single migrating neoplastic cells infiltrate the surrounding otherwise healthy brain parenchyma. Consequently, residual cancer cells inexorably remain after surgery and are responsible for tumor relapse and, as such, a poor median survival of 15 months following standard treatment protocols^2,3^.

Glioma diagnosis is usually made partly by assessing characteristics of the neoplastic cells in tissue samples, in addition to molecular investigation and, thus, may be prone to pathologist-dependent bias. Pathological evaluation of glioma tumors would benefit from additional biomarker assessment. In general, using a single protein/analyte as a disease biomarker is insufficient for an accurate and specific diagnosis; thus, there is a tendency to combine several biomarkers in multi-analyte panels to improve cancer diagnosis^4^. Indeed, there is now mounting evidence that a multi-analyte panel of biomarkers offers statistical advantages over individual biomarkers for discerning diagnostic and prognostic features across a variety of cancer states^5^.

So far, several biomarkers with prognostic and diagnostic relevance have been identified in GBM. The best-studied of which include the loss of chromosome 10q heterozygosity, presence of EGFR variant EGFRvIII^6^, and the methylation status of *MGMT* and *PTEN* promoters^7^. *MGMT* promoter methylation status is currently the most commonly used prognostic marker, as it is efficient at anticipating response to the first-line standard treatment in newly diagnosed GBM patients^8^. In terms of diagnosis, *IDH1/2* genotyping is helpful in predicting the histological grade (2016 classification) and is now systematically used to make the distinction between GBM and non-GBM tumors (IDH-mutated grade 4 astrocytomas) in the recent classification^1,9^. However, as the prognosis of glioblastoma remains poor, novel biomarkers that would refine the diagnosis and prognosis, in addition to therapeutic targets, are warranted.

Mucins are heavily glycosylated proteins, expressed at the surface of normal epithelial cells, where they form a protective shield to prevent pathogen entrance^10^. Mucins can be either secreted or membrane-anchored (transmembrane mucins). Mucins are overexpressed in several carcinomas^11^, and play distinct roles in invasion, immunomodulation, and chemoresistance^12^. In particular, mucin 4 (MUC4) contains three highly conserved EGF-like sequences, and a cytoplasmic tail (CT), strongly suggesting transducing abilities^13^. MUC4 is translated into a single polypeptide, divided at the GDPH site (Gly-Asp-Pro-His) into two subunits: the α subunit, covering the entire extracellular part, and the β subunit, the transmembrane (TM) and CT domains^11^. It is believed that MUC4 can be cleaved at the GDPH site and secreted under certain conditions to strengthen the extracellular mucous gel^14^. MUC4 is overexpressed in various carcinoma cells and has been demonstrated to interact with various EGFR family members^11,15^. However, only a few studies^16–18^ have investigated the implication of mucins in gliomas. According to the Human Protein Atlas database, MUC4 is not expressed in the healthy brain. Interestingly, MUC4 overexpression is observed in GBM patient samples and GBM cell lines. In addition, in vitro assays indicate that induced expression of MUC4 in GBM cell lines increases the proliferation and invasion abilities of the cells^16^. Moreover, a recent study found that, along with *TP53* and *IDH1*, MUC4 was one of the genes most frequently affected by single nucleotide polymorphism (SNP) in IDH-mutated (IDH-m) patients progressing towards grade 4 astrocytoma (secondary GBM; 2016 classification) suggesting the MUC4 protein involvement in gliomagenesis^19^.

In GBM, the epidermal growth factor receptor (EGFR) is the most commonly amplified gene and is overexpressed in about 60% of tissue from patients with primary GBM^20^. In the extracellular space, EGFR interacts with a variety of growth factors and ligands, such as EGF and neuregulins, triggering a wide array of cellular phenotypes, including cell migration, growth, and adhesion. Given that this receptor is commonly dysregulated in cancer, there has been enormous interest in studying EGFR-driven signalling cascades. Matrix metalloproteinases (MMPs) have also been shown to play a major role in glioma progression, with MMP9 being the most well-studied^21^. MMP9 has been previously suggested as a prognostic and diagnostic factor in glioma^22^. This protease mainly acts by inducing angiogenesis^23^, as well as by degrading collagen, thus facilitating tumor cell migration within their surrounding microenvironment^24^. Importantly, MMP9 seems to be transcriptionally regulated by EGFR signalling and involved in the regulation of key signalling pathways in glioma, such as PI3K/AKT, STAT3/5, NFkB, ERK, and SHH^25–27^. In general, EGFR interacts with members of the mucin family in cancer^11,15^, and as such, MUC4 may physically interact with EGFR in a ligand-dependent manner, leading to downstream signalling and MMP9-mediated glioma initiation and progression.

In this study, we hypothesize that the components of the EGFR/MUC4/MMP9 axis may constitute a panel of novel biomarkers and therapeutic targets for GBM. We therefore evaluate the clinical relevance of EGFR, MMP9, and MUC4 proteins for glioblastoma diagnosis and prognosis. Since grade I gliomas (pilocytic astrocytomas) are molecularly and histologically distinct from the other glioma grades and cannot evolve into higher glioma grades, we focused here on grade II (diffuse astrocytoma and oligodendroglioma), grade III (anaplastic astrocytoma and anaplastic oligodendroglioma), and grade IV (GBM) glioma (histological WHO classification)^28^. First, we investigated the protein expression, cellular localization, and co-localization of these proteins in tissue samples from patients with grade II, grade III, and grade IV gliomas as well as their association with clinicopathological features. The prognostic significance was next evaluated by analyzing the association between protein expression levels and the clinical outcome of glioma patients using Kaplan–Meier survival analysis. Finally, we explored the potential of MUC4 and MMP9 as serum biomarkers by measuring protein levels in serum samples of high-grade glioma patients and non-glioma (control) individuals. Overall, our study postulates a disturbance in the EGFR/MUC4/MMP9 signalling axis as a potential novel and rationalized biomarker panel for glioma.

## Materials and methods

### Patients and clinical samples

20 μm-sections of 60 formalin-fixed paraffin-embedded (FFPE) glioma tumors, obtained from tumor debulking surgery or biopsy, were analysed in total. 30 of them (10 grade II, 10 grade III, 10 grade IV) were obtained from the Manchester Cancer Research Centre (MCRC) Biobank (Manchester, UK) and corresponding numbers from the NovoPath Biobank Newcastle (Newcastle, UK) (60 in total) (Table S1). All diagnoses were performed by local Consultant Neuropathologist, following the 2016 WHO classification of the central nervous system tumors. 30 blood serum samples obtained from CNS tumor patients (10 non-glioma benign (controls), 10 grade III glioma, and 10 grade IV glioma tumors; see Table S2), were obtained from the MCRC biobank (Manchester, UK). Normal and Alzheimer’s Disease brain tissue, used as control for this study, was obtained from the Newcastle Brain Tissue Resource (NBTR) (Fig. S1). The serum samples had been stored at − 80 °C prior to analysis. Tables S1 and S2 (Supplementary File) indicate the relevant information for patients characteristics.

### Immunohistochemistry

Primary antibodies were first optimized and tested. Negative controls in each grade of the tissue samples were also used, following the same procedure but omission of primary antibody. Briefly, the FFPE tissue sections were dewaxed by serial incubation in xylene substitute (Sigma-Aldrich, MO, USA) and decreasing concentrations of ethanol. Antigen retrieval was then performed by boiling the slides in citrate buffer pH 6 or Tris–EDTA buffer pH 9 depending on the antibody. Endogenous peroxidases were blocked by incubation in a 3% H_2_O_2_ at room temperature for 20 min. Samples were also blocked with a 2%-BSA/10%-horse serum in Tris-buffered saline (TBS) solution for one hour at room temperature. Samples were incubated with the blocking solution containing primary antibodies overnight at 4 °C. Primary antibodies used were MUC4 (1:250 dilution, Ab60720; Abcam, Cambridge, UK), MMP9 (1:750 dilution, Ab76003; Abcam, Cambridge, UK), and EGFR (1:100 dilution; Ab52894; Abcam, Cambridge, UK). Universal probe, horseradish peroxidase, and diaminobenzidine tetrahydrochloride (A. Menarini diagnostic kit, Winnersh, UK) were used to reveal primary antibodies, as previously^29^. Pictures were taken with a Leica microscope DM75 (Leica microsystem, UK) at magnifications of 20 × and 40 ×. Haematoxylin and eosin staining was performed on a representative slide for each case to confirm grading. To avoid the inclusion of artefacts, slides were scored by the investigators, blind to clinicopathological data, rather than by automated techniques using software tools. Both the intensity and the proportion of expressing cells were considered during the analysis. Scoring was performed from 10 views at a magnification of 20 × for each slide and each marker. 1800 images were analysed in total.

### Immunofluorescence

FFPE slides were dewaxed by serial incubation in xylene substitute (Sigma-Aldrich, MO, USA) and decreasing concentrations of ethanol. Antigen retrieval was performed by boiling the slides in citrate buffer (pH 6). The slides were incubated in blocking solution (TBS-Tween 7% goat serum, 5% BSA) for 1 h at room temperature and then with each primary antibody solution containing the primary antibody diluted in antibody diluent (TBS-T, 2% goat serum, 5% BSA) at 4 °C overnight. Primary antibodies used were the following: mouse anti-MMP9 antibody (ab58803; Abcam, Cambridge, UK), rabbit anti-MMP9 antibody (ab38898; Abcam, Cambridge, UK), mouse anti-MUC4 antibody (ab60720; Abcam, Cambridge, UK), rabbit anti-EGFR antibody (ab52894; Abcam, Cambridge, UK) and rabbit anti-CD31 (SAB5700639; Sigma-Aldrich, MO, USA). Fluorescently labelled secondary antibodies used were: ab150083 goat anti-rabbit IgG H&L (Alexa Fluor® 647, pre-adsorbed; Abcam, Cambridge, UK); and ab150114 goat anti-mouse IgG H&L (Alexa Fluor® 555; Abcam, Cambridge, UK) diluted in the same antibody diluent as described. The slides were incubated in the secondary antibody solution for 1 h at room temperature and mounted with fluorescent mounting medium containing DAPI (ab104139; Abcam, Cambridge, UK), as previously^29^. Images were captured with a Leica SP8 fluorescent microscope (Leica microsystem, UK), using an 20 × objective.

### ELISA immunoassays

Enzyme-linked immunosorbent assays (ELISA) were used to assess the concentration of MMP9 and MUC4 proteins in the serum samples. Measurement of the two forms of MMP9 (92 kDa pro-form and 82 kDa activated form) was performed using the Human MMP9 Quantikine ELISA kit DMP900 (R&D systems, MN, USA). Human MMP9:TIMP-1 Complex DuoSet ELISA kit DY1449 (R&D systems, MN, USA) was used to measure MMP9:TIMP-1 complex. Detection of MUC4 was performed using the Human MUC4 ELISA kit EH1160 (Fine Bioteck co, Hubei, China). Each assay was used according to the kit manufacturer’s instructions, as previously. Optical density was measured with the Epoch 2 microplate spectrophotometer (BioTeK, VT, USA) at 450 nm with a wavelength correction set at 540 nm, made in duplicate for each sample. A standard curve for each protein was made for each assay in duplicate and was used to determine the protein concentration in each sample. The concentration of the total protein content in each sample was also determined using a Quick start Bradford assay (Bio-rad, CA, USA) in which a standard curve of bovine serum albumin (BSA) was used. Each protein concentration was calculated using the relevant standard curve for each ELISA (ng/ml) and normalized based on the total protein concentration (1 mg/ml).

### Statistical analysis

Chi^2^ test, Tukey’s multiple comparison test, and log-rank test were generated using GraphPad Prism 9 software (GraphPad software, CA, USA). The Chi^2^ test was used to evaluate the dependence between categorical variables in glioma tissue samples. One-way ordinary ANOVA followed by Tukey’s multiple comparison test was used to test whether the mean concentration of proteins in serum between the grades were significantly different. All p-values given were 2-sided and a p-value ≤ 0.05 at a 95% confidence interval was considered statistically significant. The number of months between surgery and death (if any) was used as overall survival (OS). The Kaplan–Meier method and log-rank test were used to study survival rates.

### Ethical approval and consent to participate

Sample collection was approved by the Research Ethics Boards of the respective Biobanks (Manchester Cancer Research Centre, UK and NovoPath Biobank Newcastle, UK) and the Teesside University Research Ethics Committee upon receipt of the ethical approval. Brain tissue was obtained from the NBTR, a UK Human Tissue Authority-approved research tissue depository, and ethical approval granted by the Newcastle University ethics board and the Joint Ethics Committee of Newcastle and North Tyneside Health Authority. All procedures followed The Declaration of Helsinki.

## Results

### EGFR, MUC4, and MMP9 tissue expression analysis and correlation with clinicopathological characteristics in glioma patients

#### EGFR, MMP9 and MUC4 expression in glioma FFPE tissue patient samples

**Figure 1:**
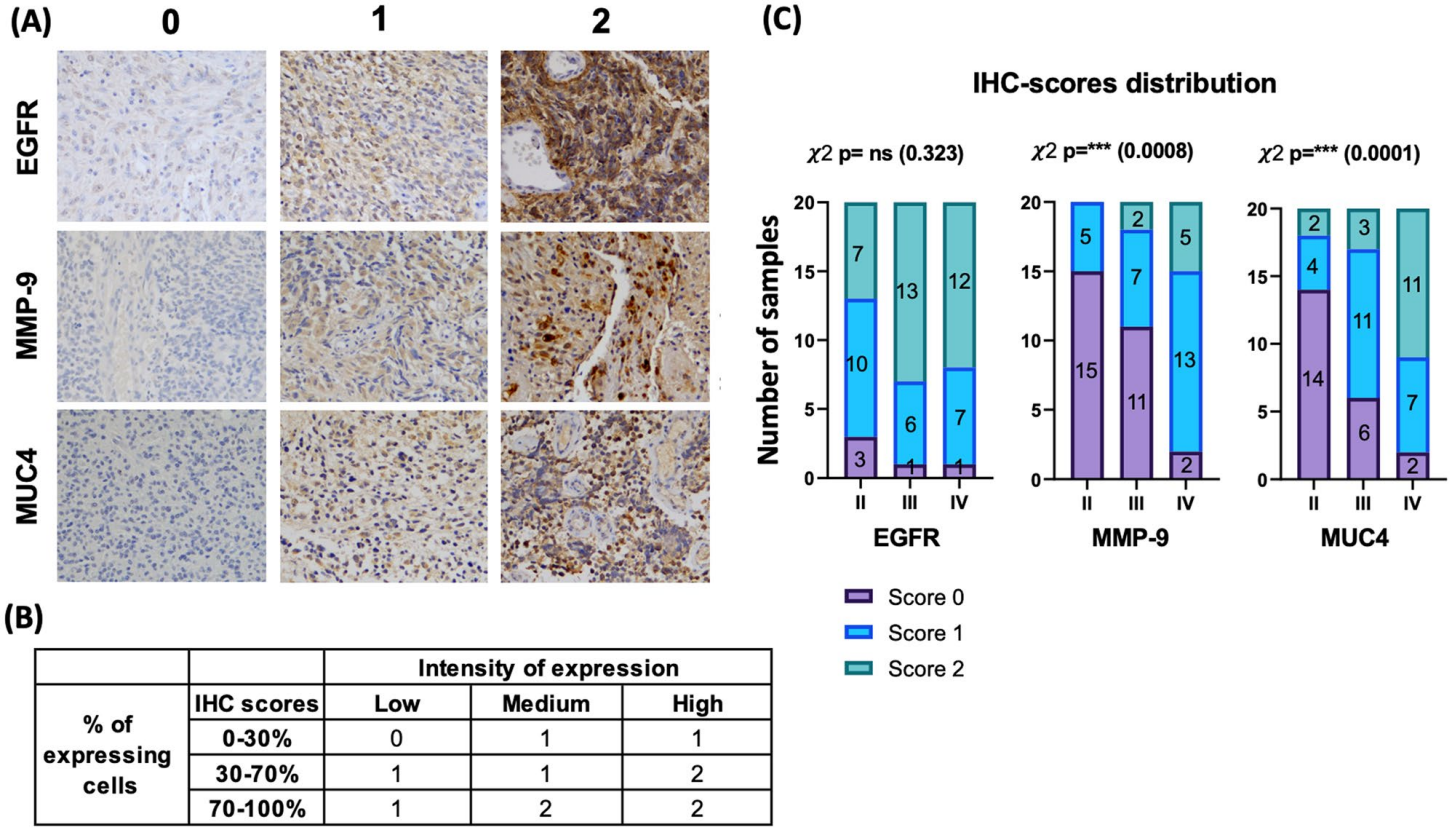
EGFR, MMP9, and MUC4 protein expression in glioma tissue biopsies. Immunohistochemistry was performed on 60 glioma tissue sections with EGFR, MMP9, and MUC4 antibodies. (**A**) The slides were categorized into three scores according to the percentage of area and intensity of staining. Score 2 represents the highest expression, magnification, 20 ×. (**B**) Table representing how the scores are distributed according to the percentage of expressing cells and the expression intensity. (**C**) Stacked bar graphs representing the distribution (contingency) of scores in grade II, grade III, and grade IV glioma for EGFR, MMP9, and MUC4 (N = 60 cases; 20 per grade) . Statistical differences were assessed with the Chi^2^ test (95% confidence interval). For EGFR, there was no statistical significance (ns = non-significant). For MMP9 and MUC4, the higher rate of expression was observed in higher glioma grades (higher levels in GBM compared to III and II). Significance is indicated on the graph as: *Denotes p ≤ 0.05, **p ≤ 0.01, and ***p ≤ 0.001.

MMP9 and MUC4 were not expressed in glial cells of non-cancerous brain tissue (Fig. S1). EGFR, MMP9, and MUC4 expression levels were assessed in FFPE tissue biopsies from 60 glioma patients (20 grade II, 20 grade III, and 20 GBM patients, following the 2016 WHO classification). The tumors were categorized into three IHC scores (0, 1, and 2) for each protein, depending on both the intensity and the percentage of expressing cells (Fig. 1A,B). MMP9 and MUC4 tissue protein expression were significantly correlated with glioma grades (Fig. 1C). Specifically, MUC4 and MMP9 high expression rate significantly increased in grade IV compared to grade III, and in grade III compared to grade II, and the association between the expression and the grades was significant for both proteins, as assessed by the Chi^2^ test (p = 0.0001 for MUC4 and p = 0.0008 for MMP9) (Fig. 1C). When comparing ‘no expression’ (score 0) versus ‘expression’ (score 1 and 2 combined), the results were similar, with the positive association between the grades and the protein expression significant for both MUC4 and MMP9 (p = 0.0003 and p = 0.0001, respectively), and combining MUC4 and MMP9 did not improve statistical significance (p = 0.0007) (Table S3). High expression of MUC4 (score 2) was observed in some grade II tumors, but not high expression of MMP9 (Fig. 1C), which was not expressed by neoplastic cells in low grade cases (grade II). Among all grades, the number of patients with high expression was higher for MUC4 than for MMP9 (26.6% vs 11.6% in total). A high number of patients expressed EGFR (91.7%), and although the number of patients showing high expression was higher in the malignant grades (III and IV) than in the lower grade (II), no statistically significant differences were observed between any of the groups (Fig. 1C).

Since IDH genotype is crucial in the current cytogenetic classification, protein tissue expression was also correlated with the IDH1 genotype (mutant or wild type), in addition to the histological grades. For MMP9 and MUC4, but not EGFR, there was a significant association between high expression and presence of wild type IDH1 (IDH-WT), with the significance greatest in MUC4 (p = 0.002 and p = 0.0007, for MMP9 and MUC4 respectively) (Table S4). This observation was expected since IDH-WT, a predictive marker of worse survival in glioma, is strongly associated with higher histological grades (90% of IDH-WT are grade IV glioma according to the 2016 WHO classification).

#### Tissue-specific localization and co-localization of protein expression

**Figure 2:**
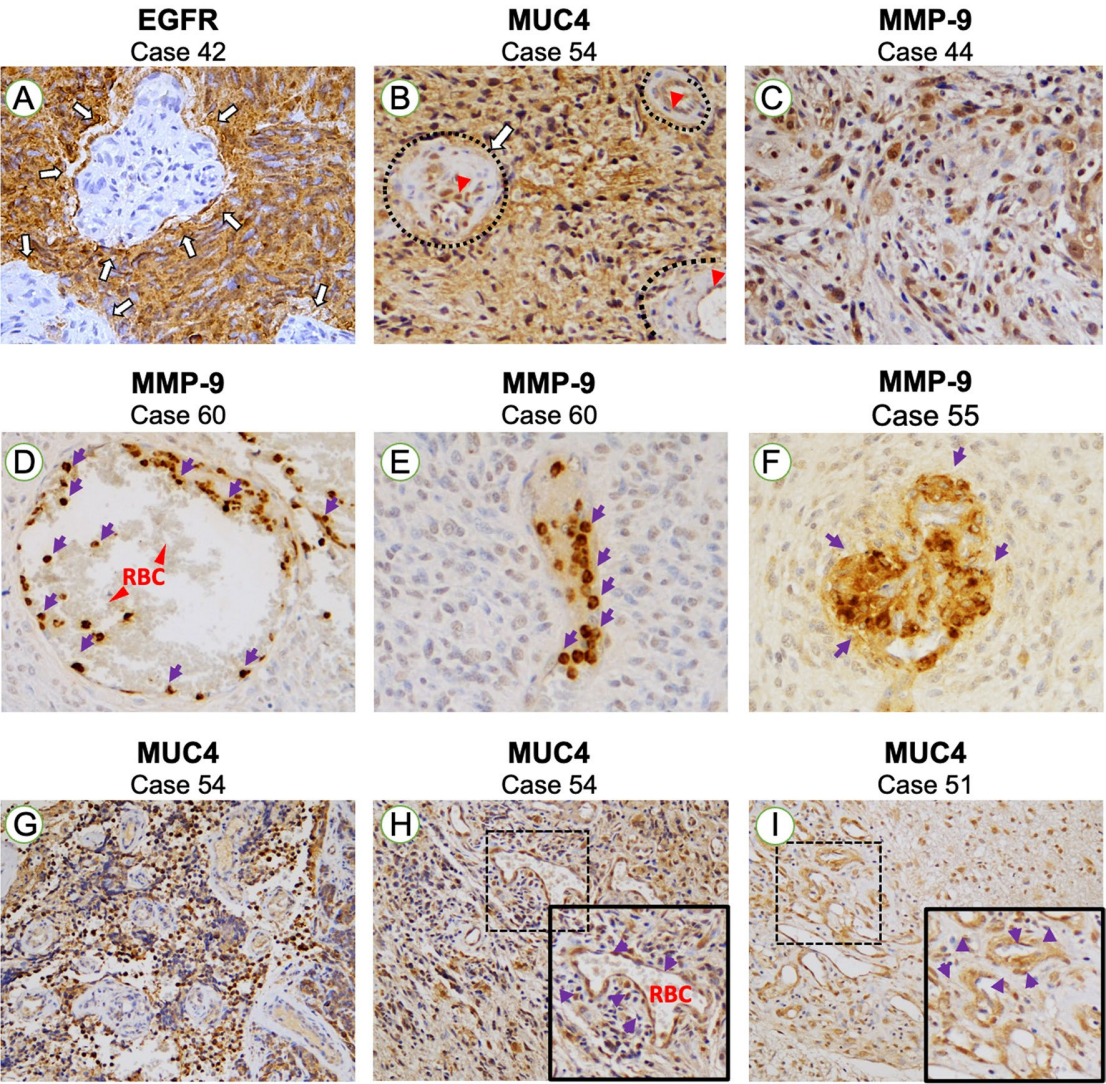
EGFR, MUC4, and MMP9 localization in GBM. (**A**) Example of one GBM case with extended cytoplasmic EGFR expression. Note the total absence of EGFR-expressing cells in the proliferative microvasculature (white arrows)**.** Magnification. 40 ×. (**B**) Example of one GBM case displaying dispersed cytoplasmic expression of MUC4. MUC4 is expressed in the endothelial wall (inner layer) of GBM microvasculature (red arrows) and in neoplastic cells, but not in the outer layers (white arrows and black dotted lines). Magnification, 20 ×. (**C**) Example of one GBM case displaying dispersed cytoplasmic expression of MMP9 in neoplastic areas showing pleomorphism. Magnification, 20 ×. (**D**, **E**) Representative images showing specific and restricted expression of MMP9 in cells located inside vessels, resembling immune cells, in one GBM patient; the surrounding neoplastic cells are negative for MMP9. Magnification, 40 ×. (**F**) Representative image showing MMP9 strong expression in microvascular proliferative structure (glomeruli-shaped) in one GBM patient. Purple arrows indicate MMP9-expressing cells; red blood cells (RBC) localization is indicated by red arrows Magnification, 40 ×. (**G**) Example of a GBM area showing expression of MUC4 in cancer cells. Magnification, 20 ×. (**H**, **I**) Images showing MUC4 expression in endothelial walls of microvascular proliferative areas (purple arrows). Two examples (from two different GBM patients) are shown. *RBC* red blood cells. Magnification, 20 ×.

In GBM patients, EGFR was variably expressed in the cytoplasm of neoplastic cells, ranging from moderate to strong expression, across the entire section. The expression was homogeneous among all neoplastic cells but not seen in visible endothelial structures (Fig. 2A). In GBM patients expressing MUC4, the protein was found in the cytoplasm of cancer cells, in a manner similar to EGFR (Fig. 2B). MUC4 was also expressed in the endothelial cells of the GBM microvasculature (Fig. 2B, red arrows). However, no expression was present in cells located within the perivascular zone between the endothelial cell wall and neoplastic cells (Fig. 2B, black dotted lines).

The expression of MMP9 in GBM patients was less dispersed and more heterogeneous than for EGFR and MUC4, with cytoplasmic and nuclear expression showing in pleomorphic cells (cells highly heterogeneous in shapes and sizes) (Fig. 2C). In addition, MMP9 was strongly and specifically expressed in nucleated cells located inside or near blood vessels, (Fig. 2D,E), representing most probably neutrophils or monocytes/lymphocytes, and that seemed to increase in number in GBM compared to low-grade samples (Fig. S2). MMP9 also seemed to be strongly expressed in a small portion of proliferative microvasculature (MVP) structures of GBM, characterized by their glomeruli-like shape (Fig. 2F). In GBM cases displaying the highest MUC4 expression, MUC4 was prominently expressed in cancer cells (Fig. 2G). In addition, the endothelial walls of some microvascular areas in GBM were MUC4-positive and showed stronger expression in comparison to the surrounding neoplastic cells (Fig. 2H–I, two representative examples shown).

**Figure 3:**
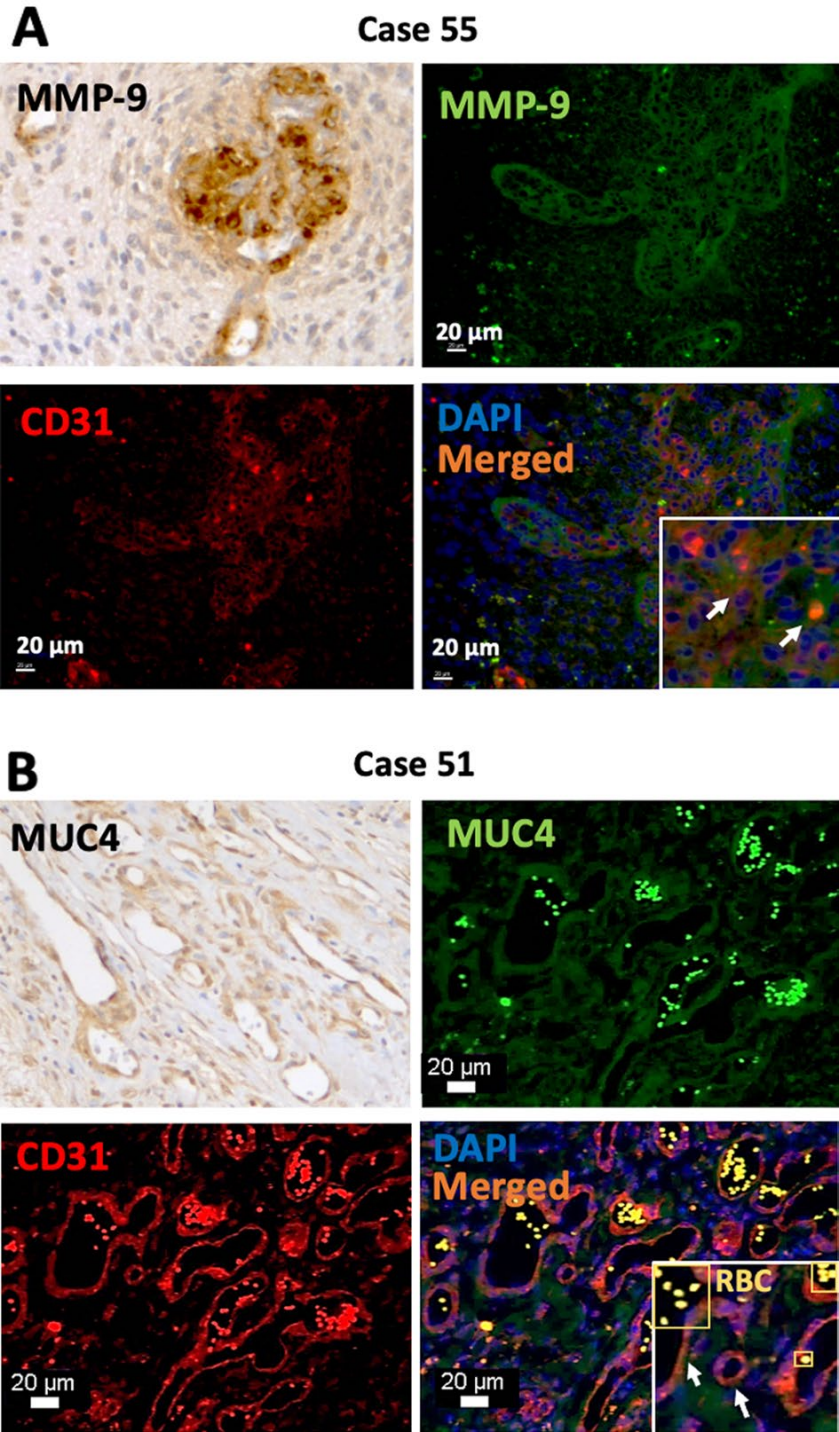
MMP9 and MUC4 expression in tumor vascular cells of glioblastoma tissues. MMP9 (**A**) and MUC4 (**B**) were expressed in vascular cells using immunohistochemistry and co-localized individually with the vascular/angiogenesis marker CD31 in double Immunofluorescence. *RBC* red blood cells, white arrows indicate co-localization. Magnification, 20–40 ×.

To confirm that MMP9 and MUC4 were expressed in the microvasculature, double immunofluorescence (IF) staining with the common pan-endothelial marker CD31 was performed (Fig. 3). The staining confirmed that structures expressing either MMP9 (Fig. 3A) or MUC4 (Fig. 3B) appeared to be CD31-positive regions (note that the strong fluorescence observed inside the blood vessels is due to the natural red blood cells’ autofluorescence properties).

**Figure 4:**
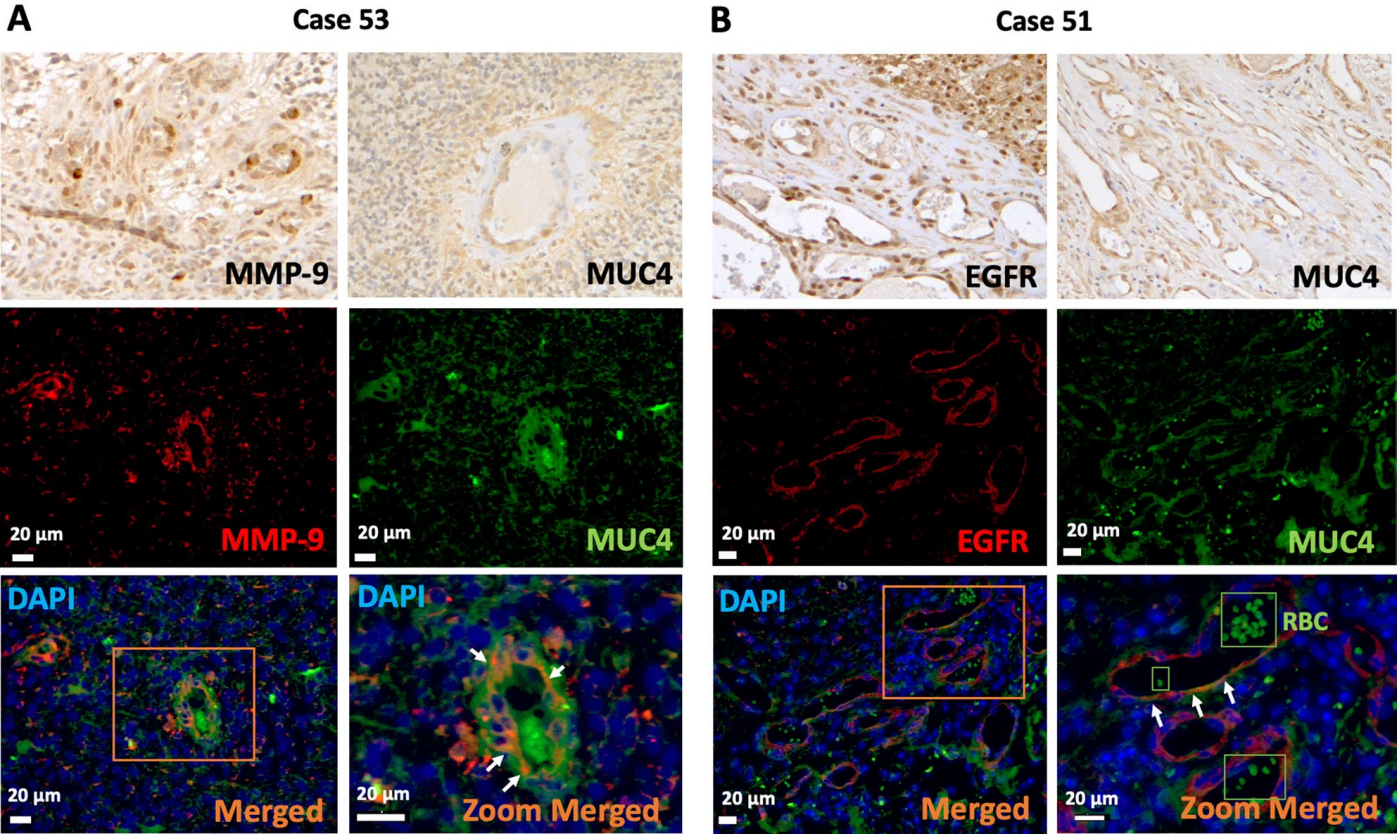
MMP9, MUC4, and EGFR co-localization in glioblastoma tissues. (**A**) MMP9 and MUC4 concomitant vascular expression in representative glioblastoma case. Co-localization of both proteins is observed in the vascular areas with double immunofluorescence. (**B**) EGFR and MUC4 concomitant expression in vascular areas in representative case of glioblastoma observed by immunohistochemistry and immunofluorescence. Co-localization of both proteins is observed with double IF. *RBC* red blood cells, white arrows indicate co-localization. Magnification, 20–40 ×.

We next tested whether MMP9 and MUC4 could be expressed together within the same MVP structures, therefore we examined selected GBM samples that showed both MMP9 and MUC4 expression in MVP structures from the IHC staining (Fig. 4A). Out of the 20 GBM patients, 8 expressed both MUC4 and MMP9 in the MVP structures. In these patients, MUC4 and MMP9 proteins seemed to be expressed together in the microvasculature (Fig. 4A). MUC4 has also been shown to interact with EGFR family members in several cancer types^11,15^. In the GBM samples, some cases also displayed a similar pattern of MUC4 and EGFR expression in MVP structures (Fig. 4B). Double IF staining revealed the co-localization of MUC4 and EGFR, again restricted to microvascular structures (Fig. 4B).

#### Association of protein expression with clinicopathological features

The clinicopathological features of the patient cohort were compared with the tissue expression for each protein separately, as well as in combination. Overall, there was no significant correlation between age or gender and expression for any of the three proteins (Table S4). For MMP9 and MUC4, but not EGFR, there was a significant association between high expression and presence of wild type *IDH* gene (p = 0.02 and p = 0.004 respectively, and p = 0.03 for MUC4/MMP9 combined, Fig. S3A). This observation is expected since IDH wild type, a marker predictive of worse survival in glioma, is more prevalent in GBM than in low-grade gliomas (90% vs 10%), when following the 2016 WHO classification^30^. Of note, the overall survival of IDH-WT patients in our cohort was worse than the one of IDH-m patients and, as expected, the difference was highly significant using the Kaplan–Meier method (p < 0.0001, Log-rank test, Fig.S3B). This result further confirms that MUC4 and MMP9 protein levels are positively associated with GBM among glioma samples.

**Figure 5:**
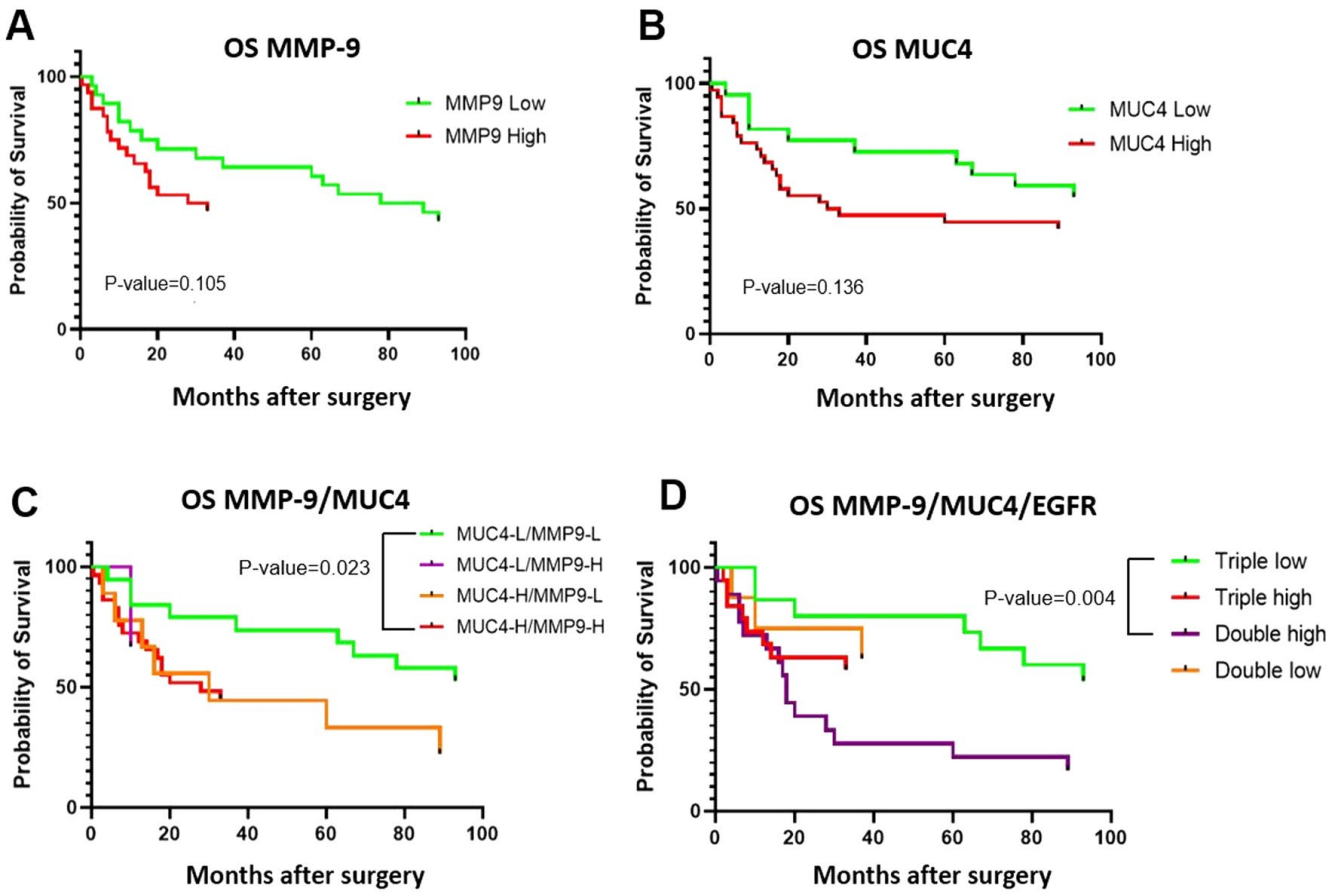
Survival analyses in glioma patients. Comparison of overall survival (OS) (**A**) between two MMP9 tissue expression groups (low and high) in glioma patients. High expression of MMP9 predicts worse outcome than low expression, but the difference is not significant; (**B**) between two MUC4 tissue expression groups (low and high) in glioma patients. High expression of MUC4 predicts worse outcome than low expression, but the difference is not significant. (**C**) MUC4-MMP9 combined high expression significantly predicts worse survival in glioma patients as assessed by the log-rank test for trend (p = 0.018). MUC4-L/MMP9-L patients have better survival than MUC4-H/MMP9-H patients as assessed by log-rank test (Mantel Cox, p = 0.023). (**D**) MUC4/MMP9/EGFR expression significantly predicts worse survival in glioma patients. Triple low patients (with low expression for the three markers) have better survival than double high patients (with high expression of two out of the three markers) as assessed by log-rank test (Mantel Cox, p = 0.004).

The clinical outcome was compared between patients with a low level of tissue expression and patients with a high level of tissue expression, using Kaplan–Meier survival analysis. The three IHC-scores were categorized into two groups: High and Low expression, and the clinical outcome (overall survival (OS)) were compared for each protein. The EGFR-high group comprised score-2 and the MMP9-high and MUC4-high groups comprised score-1 and score-2. This decision was made to compare groups of similar size. When investigated alone, MMP9-High and MUC4-High expression groups tended to indicate worse prognosis than MMP9-Low and MUC4-Low groups, respectively, although the difference was not significant (p = 0.105 for MMP9; p = 0.136 for MUC4) (Fig. 5A,B). Moreover, no difference was observed for EGFR between the two groups (Fig. S4).

We next looked at the protein expression and correlation with clinical outcomes as multi-analyte panels. Clinical outcomes were compared by combining MMP9 and MUC4 proteins with and without EGFR (Fig. 5C,D). For MMP9 and MUC4 combined, four groups were generated for the pair of proteins containing the different combinations of expression: Low MMP9 expression–Low MUC4 expression (L–L), High–High (H–H), and Low–High/High–Low (L–H and H–L). Clinical outcomes demonstrated significant differences when both proteins MMP9/MUC4 were combined (Fig. 5C). When associating MMP9 with MUC4, the L–L group displayed the best outcome and the H–H group the worst (Fig. 5C) and this trend was significant (p = 0.018 for OS, Log-rank test). More specifically, the difference between the L–L group and the H–H group was significant (p = 0.023, Mantel–Cox Log-rank test) (Fig. 5C).

Then, all three proteins were combined (Fig. 5D) and four groups were generated: “triple low” contained patients with low expression for the three proteins; “triple high” patients with high expression for the three proteins; “double low” patients with low expression for two proteins out of three; and “double high” patients with high expression for two proteins. The MUC4/MMP9/EGFR combined low expression significantly predicted better survival in glioma patients in comparison with the ‘double high’ group and the difference was again more significant than the previous analysis that combined MMP9 and MUC4 only (without EGFR) (p = 0.004, Log-rank). Therefore, adding EGFR was useful to improve the accuracy of outcome prediction. The biggest difference was observed between ‘triple low’ and ‘double high’ (p-value = 0.004) and not between ‘triple low’ and ‘triple high’ (p-value = 0.15). However, this could be due to the smaller size of ‘triple high’ (n = 8 for OS and n = 7 for PFS) in comparison with the ‘double high’ group (n = 15 for OS and n = 11 for PFS).

### Serum protein level measurements of MMP9 and MUC4 in glioma patients

**Figure 6:**
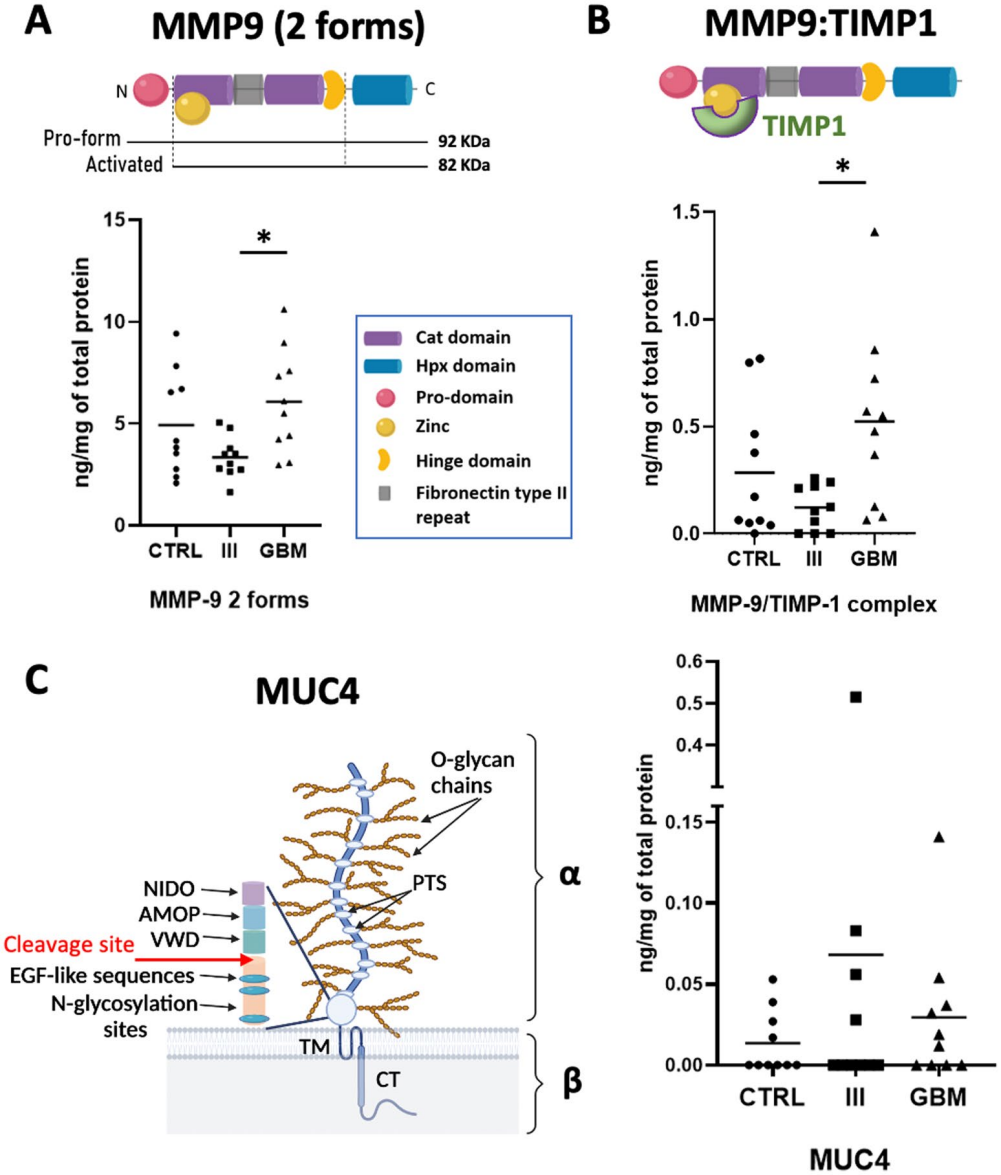
MMP9 and MUC4 protein serum levels determined by ELISA immunoassays. Scatter graphs showing two forms of MMP9 (active form and pro-form) (**A**), MMP9 bound to TIMP1 (**B**), and MUC4 (**C**) individual concentration values (with means) of non-glioma (CTRL: controls), glioma grade (III), and glioblastoma (GBM) patients. N = 10 for each group (30 patients in total). MMP9 protein levels (ng/ml) and MUC4 protein levels (ng/ml) measured in serum, were normalized based on the total protein; results are given as ng/ml per mg/ml of total protein. The different forms of MMP9 measured in each assay are represented in (**A**) and (**B**), and MUC4 structure and cleavage site is represented in (**C**). Bars represent protein mean levels. MMP9 two forms protein levels as well as the MMP9:TIMP1 complex are significantly higher in the GBM than in grade III group (Tukey’s multiple comparison test, p = 0.022 and 0.018, respectively). MUC4 values are represented on a stretched Y axis in (**C**). Higher concentrations of MUC4 are found in malignant glioma grades compared with controls but the difference does not reach significance. *NIDO* Nidogen-like domain, *AMOP* Adhesion-associated domain, and *VWD* Von Willbrand factor domain.

We further investigated the potential use of MMP9 and MUC4 proteins as serum biomarkers in glioma patients. Since we previously found that MMP9 and MUC4 were upregulated in the tissue of patients with higher glioma grades (Fig. 1), we further assessed the protein levels of these two proteins in the serum of 30 patients (10 benign non-glioma tumors (controls), 10 glioma grade III, and 10 glioma grade IV) using ELISA immunoassays. The ELISA assay for MMP9 detected two forms of MMP9 found in blood (the 92 kDa pro-form and the 82 kDa activated form, see illustration in Fig. 6A). The mean MMP9 protein level was more elevated in GBM compared to both grade III and control samples. Only the difference between GBM and grade III was significant (p = 0.022, Tukey’s multiple comparison) (Fig. 6A,B). Since MMP9 may also be bound to its inhibitor TIMP1 (see illustration in Fig. 6B), and this complex may be represented with diagnostic value^31^, we next measured the levels of the MMP9:TIMP1 complex in the same samples. We found that the trend for MMP9:TIMP1 complex was similar to the free MMP9 forms, with a significant increase of the MMP9:TIMP1 complex mean protein level in GBM compared to grade III (Tukey’s multiple comparison test, p = 0.018) (Fig. 6B). The percentage of MMP9 protein bound to its inhibitor was low in all cases, with 5% of MMP9 bound to TIMP-1 in GBM on average, twice what was observed in controls and grade III (2.7% and 2.4%, respectively) (Fig. 6B).

Difference of MUC4 protein level between grade III and IV was not significant (p-value = 0.64). MUC4 serum mean protein levels tended to be higher in the malignant glioma grades (grades III and IV) compared to the control samples, but the difference was not statistically significant (p-value = 0.42 and 0.92, respectively) (Fig. 6C). This could be due to the low number of patients and future studies are needed in a larger cohort of samples.

## Discussion

MUC4 is involved in carcinogenesis and may constitute a biomarker for several cancers including pancreatic ductal^10^, lung^32^, and oral squamous cell carcinoma^33^. It has also been demonstrated that MUC4 is overexpressed in GBM tissues and cell lines^16^. However, to the best of our knowledge, the association between MUC4 and glioma progression in clinical samples has not been studied so far. The interaction between MUC4 and EGFR at the surface of cancer cells contributes to carcinogenesis^11,15^, while MMP9 expression, whose involvement in glioma progression has been well characterized^21^, is triggered by EGFR activation in several glioma studies^25–27^. We therefore looked at their protein expression in glioma patients to further elucidate their individual diagnostic and prognostic value. MMP9 and MUC4, but not EGFR, protein expression is correlated with increased glioma histological grades (Fig. 1) and IDH-WT genotype. Given that MMP9 has been proposed as a prognostic and diagnostic marker in glioma^22,25,34^, our observations that MUC4 was at least as efficient as MMP9 in discrimination between grades indicates it may act as a biomarker in glioma diagnosis (Fig. 1C).

EGFR has been shown to be overexpressed, amplified, and/or mutated in glioma. Its prognostic and diagnostic value, as well as its therapeutic targeting, have led to contradictory results^35^. Here, we found that EGFR was expressed in most of our samples (91.7%) and overexpressed in around 60% of GBM patients, therefore failing to discriminate among glioma grades (Fig. 1C). This implies EGFR, at least individually, may not be a powerful diagnostic biomarker in glioma. However, using EGFR amplification or the expression of specific EGFR variants could yield different results. We found EGFR highly expressed in the cytoplasm of neoplastic cells but absent from a high number of visible microvascular structures. It has been shown that GBM cells with stem cell abilities can transdifferentiate into endothelial-like cells that could contribute to the tumor microvasculature, a process termed “vasculogenic mimicry”, which was observed during in vitro and in vivo, but not in clinical studies^36,37^. This is in line with our study since we found no EGFR expression within most visible microvasculature (Fig. 2A). However, we also found EGFR expression in some microvasculature structures in GBM when looking more in depth at the IHC analysis. This could imply that some microvascular structures emerge from existing endothelial cells (EGFR-negative) while others are the results of vascular mimicry from neoplastic cells (EGFR-positive), the latter being hard to identify as the strong expression of EGFR may mask the microvasculature shape using conventional IHC staining. However, additional studies are needed to confirm this hypothesis.

Our results indicate that MMP9 was restricted in cells of haematopoietic origin, located near or inside the blood vessel lumen. Given their morphological characteristics, these cells were most likely neutrophils or lymphocytes (Fig. 2D,E). High MMP9 expression in normal condition is observed in cells residing mainly in the bone marrow and lymph nodes (Human Protein Atlas). Neutrophil-derived MMP9 has a role in migration through basement membrane, vessel angiogenesis, and cancer progression. On the other hand, MMP9 has also been found expressed by monocytes and lymphocytes in some cancers, such as in colorectal cancer, where the expression correlated with angiogenesis and necrosis areas^38^. A very recent study showed that MMP9 was expressed mainly in neutrophils and dendritic cells located inside vessels in newly diagnosed GBM^39^, which is in total accordance with our study. Our results indicate that MMP9-expressing cells could increase in number during glioma progression, as observed in other types of cancer^40,41^. We also found strong expression of MMP9 in cells composing the proliferative microvasculature of some areas in GBM samples (Fig. 2F), in accordance with previous studies. MMP9 is a well-characterized angiogenic mediator expressed in the microvasculature of GBM^23,42,43^, where it specifically induces basement membrane degradation and increases VEGF bioavailability, enabling angiogenesis^44^. Our results demonstrate that MMP9 was strongly expressed in some microvasculature foci of GBM, while its expression in the vascular areas in lower-grade gliomas was not observed. This supports MMP9 being involved in angiogenesis and proliferation of neoplastic vascular cells specific to GBM. Microvascular proliferation, which forms the microvasculature, is observed in GBM, but not grade II and III glioma^45^.

MUC4 was expressed homogeneously within the entire tumor parenchyma, mostly in the tumor cell cytoplasm, with less restricted localization than MMP9 (Fig. 2C). In these tumors, MUC4 was expressed in the microvasculature (Fig. 2H–I). A previous study showed MUC4 expression in human blood vessels, indicating a role in protection against pathogen adhesion^46^ and studies in mice have suggested a role in angiogenesis^47,48^. A recent study has demonstrated that *MUC4* is one of the most frequently mutated genes, along with *TP53* and *IDH1*, in IDH-m anaplastic astrocytoma patients progressing towards grade 4 astrocytoma (2021 WHO classification)^19^. In addition to being expressed in the microvasculature, our results showed that MUC4 total expression increases during glioma progression, with higher protein expression in GBM compared to lower grades (grades II and III). Overall, this could suggest that, like MMP9, MUC4 could be involved in glioma progression as well as in angiogenesis/microvascular proliferation in GBM and could serve as a potential therapeutic target. Finally, we found that MMP9 and MUC4, as well as EGFR and MUC4, co-localized in the microvasculature (Fig. 4), suggesting EGFR and MUC4, both expressed on the cell surface, could interact with, and trigger MMP9 expression. Given that this study supports the combined potential of MUC4 and MMP9 as biomarkers for diagnosis in glioma tissue, future studies underlying the detailed mechanistic underpinnings using in vitro assays and preclinical animal models is warranted.

The results showed that when used individually, MMP9 and MUC4 tissue expression can predict clinical outcome in glioma patient diagnosis, which is not the case with EGFR. Also, the correlation with clinicopathological characteristics indicated no significant clinical association between overall survival (OS), and MUC4 or MMP9 individual expression. However, the difference observed for the OS was only significant when combining MMP9 and MUC4 together, and even more when adding EGFR (Fig. 5). This suggests that these proteins together could be useful as biomarkers to diagnose glioma in tissue biopsy. However, future studies in larger cohort should additionally take into consideration different parameters, such as MGMT promoter methylation, extent of tumor resection, tumor enhancement, and treatment.

As already indicated, MUC4 and MMP9 can be detected and measured in tissue biopsies systematically collected in patients diagnosed with glioma. However, their detection as non-invasive serum biomarkers in serum samples would be beneficial due to the less invasive nature, and perhaps the greater representation of whole tumor characteristics. Using ELISA immunoassays, our study showed that the mean protein MMP9 level was significantly elevated in the serum of GBM patients compared with the serum of grade III patients (Fig. 6A), suggesting it may aid discrimination between these two grades, although the difference with the control group was not significant. It has been shown that grade III and grade IV glioma patients with no sign of radiographic progression had lower MMP9 levels in their serum than glioma patients with active disease, as well as a transient increase in the serum after resection^49^. The current study did not consider such radiographic evidence, which could constitute a limitation. Finally, we tested the level of the MMP9:TIMP1 complex in serum samples since it may also have diagnostic/prognostic value. MMPs can bind to endogenous inhibitors and interestingly, the ratio between their free and bound forms in serum may be used for diagnostic and prognostic purposes^31^. We found a similar trend for the MMP9:TIMP1 complex and free MMP9 forms (Fig. 6A,B), as the percentage of MMP9 protein bound to its inhibitor was low in all the cases, indicating it mostly exists in the free form. However, the ratio of MMP9 bound to its inhibitor, TIMP1, was slightly more elevated in GBM than in lower grade and control group, which could have a diagnostic relevance. This aligns with previous findings showing TIMP1 level is more elevated in GBM patients than in control individuals^50^.

Finally, MUC4 was also detected in the serum, albeit at lower levels. MUC4 is a transmembrane mucin that can also be secreted in order to facilitate mucus production in some conditions, including malignancies, similar to other mucins^14,51^. Our results showed that albeit MUC4 levels were low, they were only minimally elevated in higher glioma grades (III and IV) compared to controls, although the difference was not significant, which needs confirmation in a larger cohort of patients (Fig. 6C). There are currently several glycosylated proteins used as cancer biomarkers and some of them are mucins^52–55^, which often represent transmembrane proteins secreted into the circulation after cleavage, thus presenting lower levels in serum compared to other proteins. However, they still provide helpful diagnostic information, justifying the combination with other cancer biomarkers.

In conclusion, MMP9 and MUC4 are both expressed in the microvasculature of glioblastoma, indicating both proteins may be involved in angiogenesis and microvascular proliferation (MVP). In addition, the strong correlation of MUC4 and MMP9 expression with clinicopathological stage suggests both could serve as useful tissue diagnostic biomarkers in glioma patients. Moreover, the combined MUC4/MMP9/EGFR axis expression pattern could refine the prognosis accuracy. These potential biomarkers could potentially be used in liquid biopsies, which would represent an important non-invasive strategy, though further studies must be conducted to confirm the relevance of MUC4 in this capacity.

## Supplementary Information

Supplementary Information.

## Data Availability

The datasets generated and/or analysed during the current study are available from the corresponding author on reasonable request.

## Abbreviations

CNS: Central nervous system
EGFR: Epidermal growth factor receptor
ELISA: Enzyme-linked immunosorbent assay
ERK: Extracellular signal-regulated kinase
FFPE: Formalin-fixed paraffin-embedded
GBM: Glioblastoma
IDH: Isocitrate dehydrogenase
IF: Immunofluorescence
IHC: Immunohistochemistry
MGMT: O-6-Methylguanine-DNA methyltransferase
MMP9: Matrix metalloproteinase 9
MUC4: Mucin 4
MVP: Microvascular proliferation
NFkB: Nuclear factor kappa B
OS: Overall survival
PTEN: Phosphatase and tensin homolog
SHH: Sonic hedgehog
SNP: Single nucleotide polymorphism
STAT3/5: Signal transducer and activator of transcription 3/5
TIMP1: Tissue inhibitor of matrix metalloproteinases 1
TP53: Tumor protein 53
VEGF: Vascular endothelial growth factor
