# Supplementary material for: The diagnostic and prognostic potential of the EGFR/MUC4/MMP9 axis in glioma patients
Source: Sci Rep. 2022 Nov 18;12:19868. doi: 10.1038/s41598-022-24099-4 (PMC9674618; doi:10.1038/s41598-022-24099-4)
Supplement: Supplementary file 1 — Supplementary Information. [file 41598_2022_24099_MOESM1_ESM.docx]

**Supplementary File**

**Table S1.** **Fixed brain tumor tissue samples, patient characteristics.** Yrs = years; OS = overall survival; XRT = radiotherapy; PCV = procarbazine, lomustine (CCNU), and vincristine; TMZ = temozolomide; m = mutated; WT = wild type. [Note that the grading for the samples used in this study followed the 2016 WHO classification that is based on histological characteristics. In the 2021 WHO classification, which is mostly based on cytogenic features, almost all IDH-Wild Type (WT) glioma are classified as GBM, even if lacking GBM histological hallmarks; no IDH-mutated (m) astrocytoma are classified as diffuse, anaplastic, or GBM, but instead grade 2, 3, or 4 IDH-m astrocytoma; and oligodendroglioma are all IDH-m].

**Table S2.** **Blood serum samples, patient characteristics.** Yrs = years; OS = overall survival; PFS = progression-free survival; XRT = radiotherapy; PCV = procarbazine, lomustine (CCNU), and vincristine; TMZ = temozolomide; m = mutated; WT = wild type. [Note that the grading for the samples used in this study followed the 2016 WHO classification that is based on histological characteristics. In the 2021 WHO classification, which is mostly based on cytogenic features, almost all IDH-Wild Type (WT) glioma are classified as GBM, even if lacking GBM histological hallmarks; no IDH-mutated (m) astrocytoma are classified as diffuse, anaplastic, or GBM, but instead grade 2, 3, or 4 IDH-m astrocytoma; and oligodendroglioma are all IDH-m].


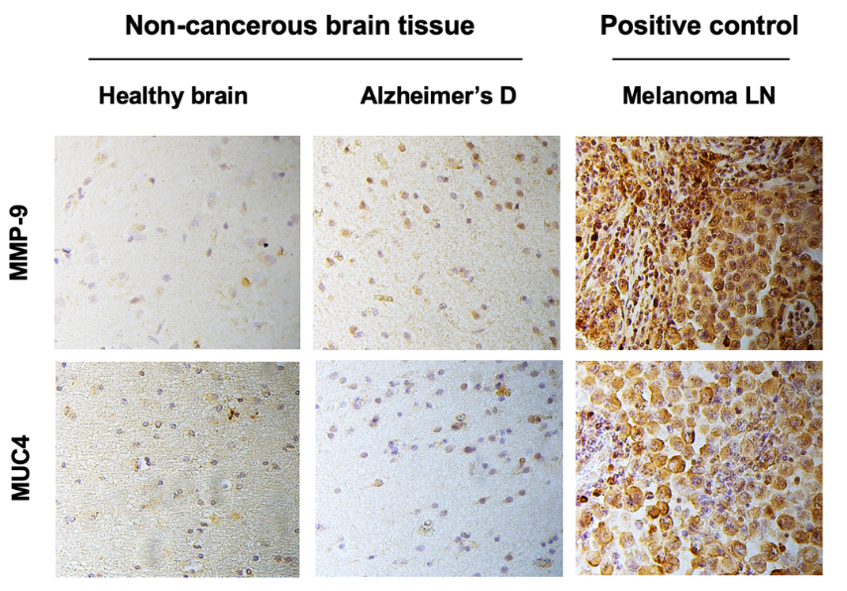


**Figure S1 Protein expression in control brain tissues.** MMP9 and MUC4 protein expression was assessed by immunohistochemistry. MMP9 and MUC4 showed very low to undetectable expression in non-cancerous brain tissues (healthy brain and Alzheimer’s Disease brain). Magnification, 40X.

**
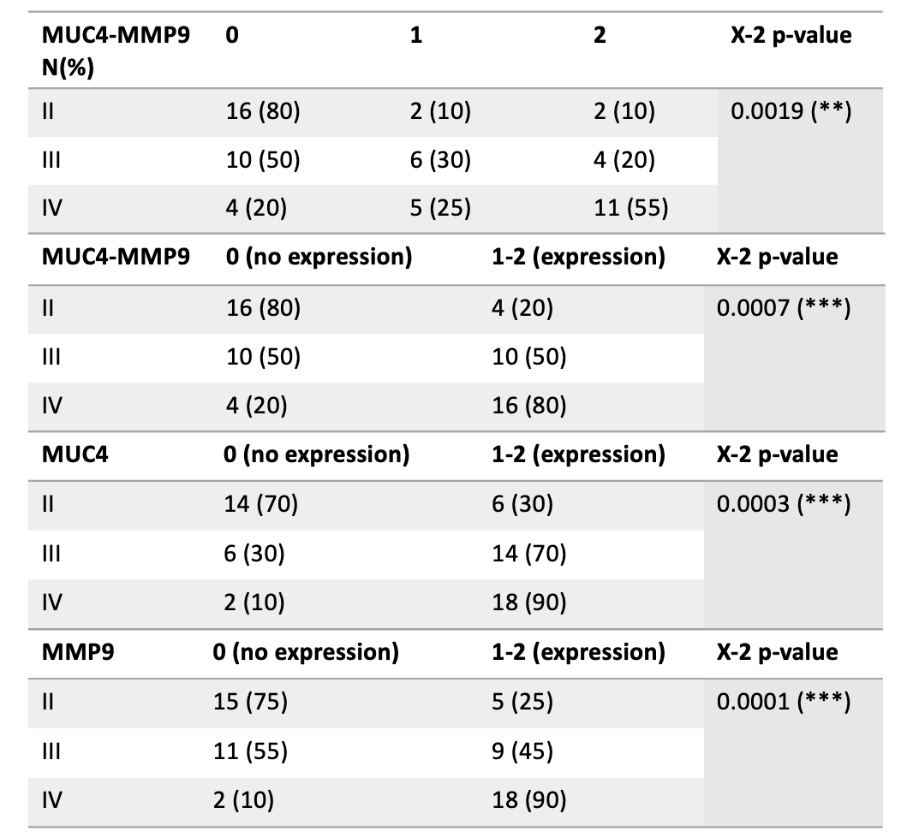
**

**Table S3** **Association between MMP9 and MUC4 IHC-scores combined and glioma grades.** Statistical differences were assessed with the Chi^2^ test (95% confidence interval). Significance is indicated as: * denotes p≤0.05, ** p≤0.01, and *** p≤0.001. The combined high expression of MMP9 and MUC4 was significantly and positively associated with the grades (p=0.0019; and p=0.0007 when comparing ‘no expression’ with ‘expression’). MUC4 and MMP9 high expression were significantly and positively associated with the grades when comparing ‘no expression’ with ‘expression’ status (p=0.0003 and 0.0001, respectively).

**
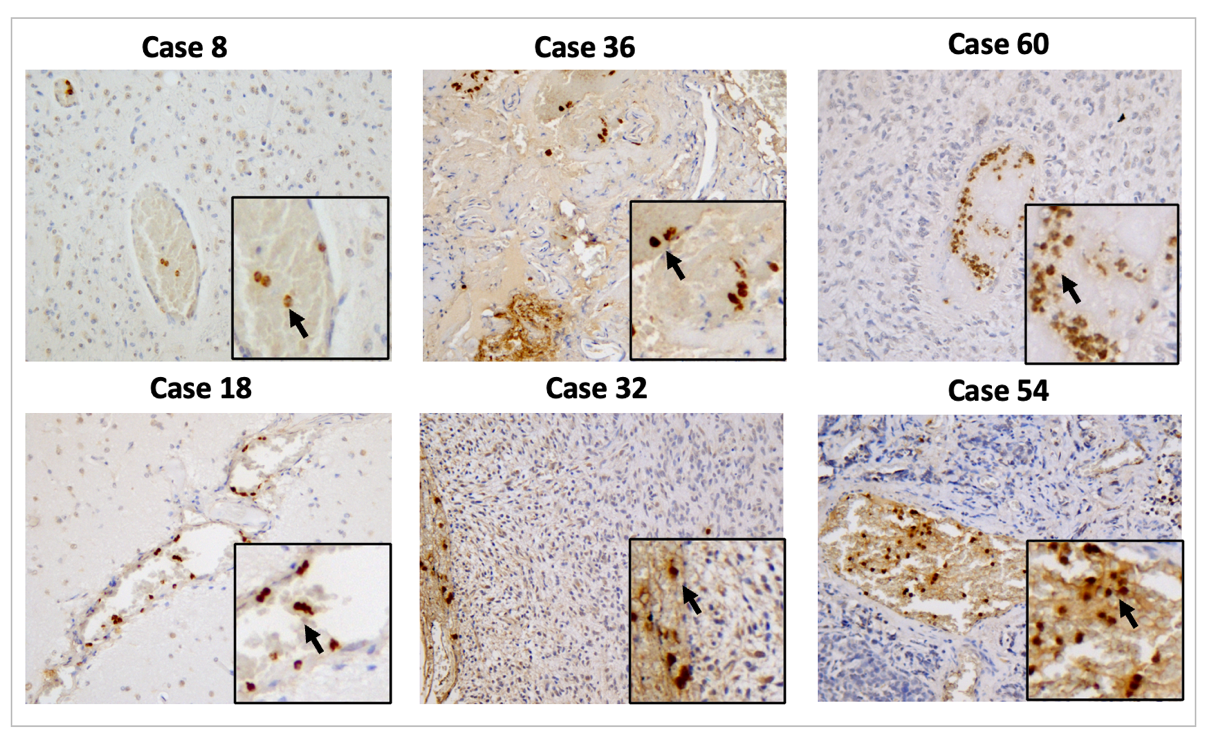
**

**Figure S2 MMP9-expressing haematological cells in glioma.** MMP9 was expressed in hematological cells inside vessels in glioma (indicated by black arrows), as assessed by immunohistochemistry. The number of these cells increase in higher grades. 2 examples shown in grade II (case 8 and 18), grade III (case 36 and 32), and GBM (case 60 and 54). Magnification, 20X.

**Table S4.** **Association between tissue protein expression and patients’ characteristics.** Statistical differences were assessed with the Chi^2^ test (95% confidence interval). Significance is indicated as: * denotes p≤0.05, ** p≤0.01, and *** p≤0.001. MMP9 and MUC4 lower expression was significantly associated with IDH-mutated patients (p=0.002 and 0.0007, respectively).

**
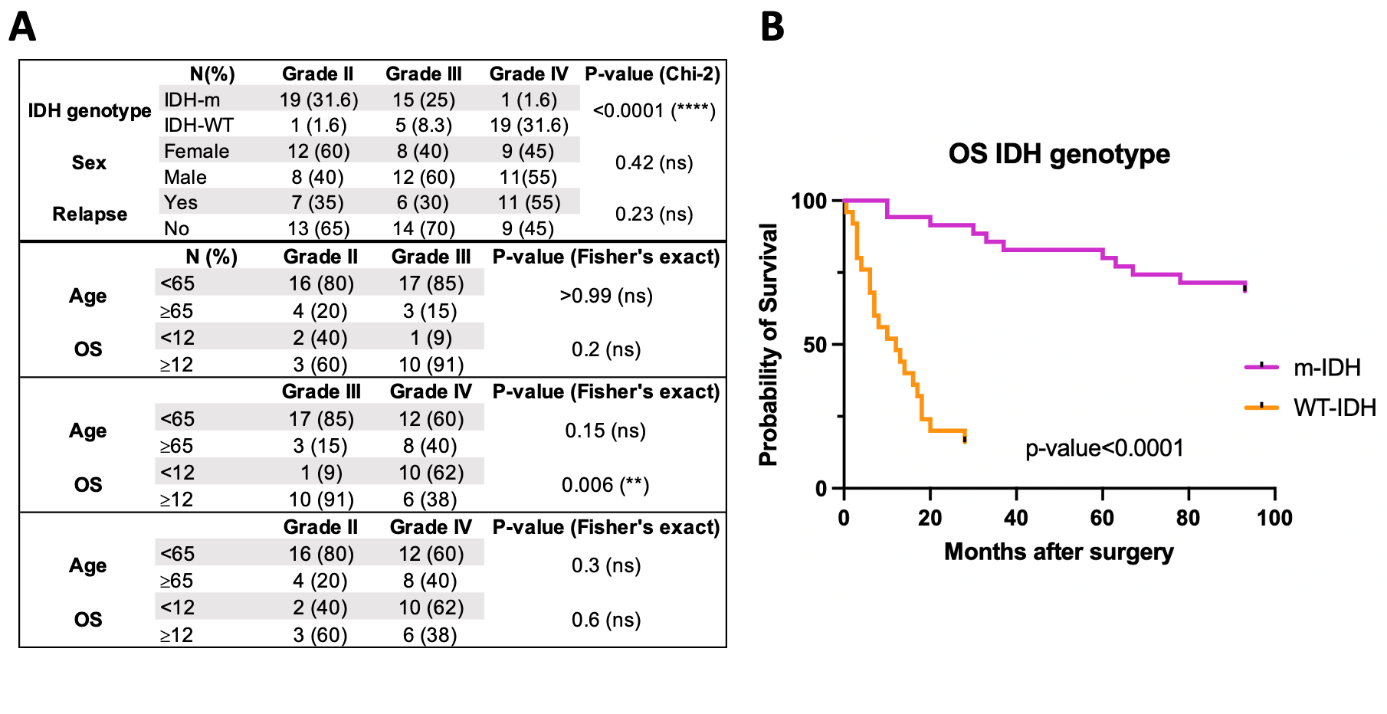
**

**Figure S3 Association between patients’ characteristics. (A)** Association between IDH genotype, sex, and relapse, with glioma histological grades was assessed by chi-square test; and association between age and overall survival (OS) with glioma grades was assessed by Fisher’s exact test. *** indicates a p-value ≤0.001; ** indicates a p-value ≤0.01; * indicates a p-value ≤0.05; ns = non-significant. The association between wild-type-IDH and higher glioma grades was strongly significant (****) and OS was significantly lower in grade IV patients compared with grade III patients (**). **(B)** Overall survival (OS) curves of mutated-IDH (m-IDH) and wild-type-IDH (WT-IDH) patients were generated with the Kaplan-Meier method. The OS of WT-IDH patients was significantly lower than the one of m-IDH patients as assessed by the Log rank test (p<0.0001).


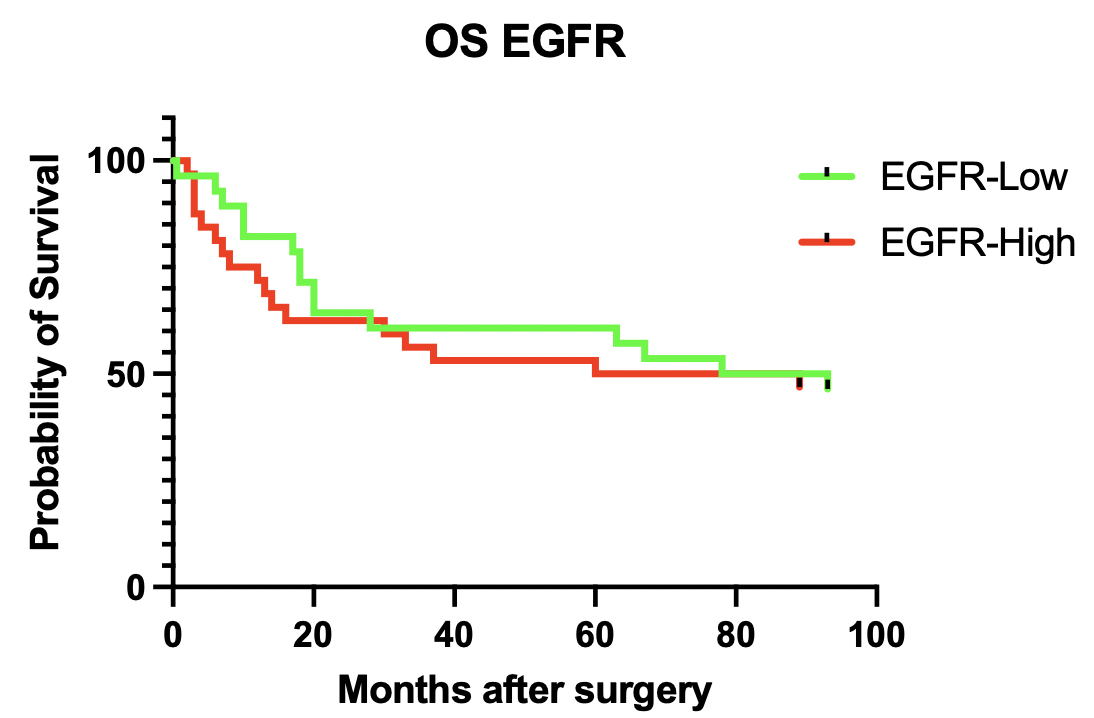


**Figure S4** **Association between patients’ survival and EGFR tissue expression.** Comparison of overall survival (OS) in all glioma patients using the Kaplan-Meier method between two tissue expression groups (low and high) for EGFR. There is no difference between the two groups (p-value = 0.63).
